# Major Risk Factors for Mortality in Elderly and Non-Elderly Adult Patients Emergently Admitted for Blunt Chest Wall Trauma: Hospital Length of Stay as an Independent Predictor

**DOI:** 10.3390/ijerph19148729

**Published:** 2022-07-18

**Authors:** Guy Elgar, Abbas Smiley, Rifat Latifi

**Affiliations:** 1 Westchester Medical Center, School of Medicine, New York Medical College, Valhalla, NY 10595, USA; 2 College of Medicine, University of Arizona, Tucson, AZ 85724, USA; latifi@surgery.arizona.edu; 3 Ministry of Health, 10000 Pristina, Kosovo

**Keywords:** chest wall trauma, thoracic trauma, hospital length of stay, respiratory disease, burns, white blood cell disease, coagulopathy, liver disease, age, in-hospital mortality

## Abstract

**Background:** Blunt thoracic trauma is responsible for 35% of trauma-related deaths in the United States and significantly contributes to morbidity and healthcare-related financial strain. The goal of this study was to evaluate factors influencing mortality in patients emergently admitted with the primary diagnosis of blunt chest wall trauma. **Methods:** Adults emergently admitted for blunt chest trauma were assessed using the National Inpatient Sample Database, 2004–2014. Data regarding demographics, comorbidities, and outcomes were collected. Relationships were determined using univariable and multivariable logistic regression models. **Results:** In total, 1120 adult and 1038 elderly patients emergently admitted with blunt chest trauma were assessed; 46.3% were female, and 53.6% were male. The average ages of adult and elderly patients were 46.6 and 78.9 years, respectively. Elderly and adult patients both displayed mortality rates of 1%. The regression model showed HLOS and several comorbidities as the main risk factors of mortality Every additional day of hospitalization increased the odds of mortality by 9% (OR = 1.09, 95% CI = 1.01–1.18, *p* = 0.033). Mortality and liver disease were significantly associated (OR = 8.36, 95% CI = 2.23–31.37, *p* = 0.002). Respiratory disease and mortality rates demonstrated robust correlations (OR = 7.46, 95% CI = 1.63–34.11, *p* = 0.010). Trauma, burns, and poisons were associated with increased mortality (OR = 3.72, 95% CI = 1.18–11.71, *p* = 0.025). The presence of platelet/white blood cell disease correlated to higher mortality. (OR = 4.42, 95% CI = 1.09–17.91, *p* = 0.038).

## 1. Introduction

Blunt thoracic injuries result in a significant amount of admissions, disability, and mortality throughout the world. Previous studies have shown that 35% of trauma-related deaths in the United States are the result of blunt thoracic trauma [1]. Dogrul et al. stated that one out of four trauma patients dies due to thoracic injuries. High-speed vehicle accidents result in blunt thoracic trauma in approximately 60% of patients with polytrauma and have a mortality rate of 20–25% [2]. In addition, blunt chest wall injuries greatly stress healthcare systems, particularly trauma centers and acute care settings where costs are estimated to be greater than USD 670 billion annually in the United States [3]. These results demonstrate the prevalence and disproportionately high acuity of blunt chest wall trauma. The research has found that 58% of trauma-related deaths are preventable [4]. This finding stresses the importance of identifying accurate prognostic parameters for patients with blunt thoracic trauma. Currently, there are numerous scoring methods involved in prognosticating patients with blunt chest wall trauma. These include but are not limited to the injury severity score (ISS), new ISS, trauma and injury score, and thorax trauma severity score. Although many studies have attempted to quantify the mortality-predicting capacity of these tools in the setting of blunt chest trauma, their results have been inconsistent [5]. These inconsistencies highlight the need for further exploration of mortality factors involved in blunt chest wall injuries.

Assessment of the current parameters incorporated into scoring mechanisms reveals several recurring themes. The majority of scoring tools incorporate pulmonary disease, rib fractures, vital signs, and age as mortality factors. While these characteristics provide clinicians with useful information, they are limited in their utility. Further analysis of patient demographics, comorbidities, and clinical treatment patterns involved in blunt chest wall traumas are essential to identifying novel parameters to be used for improved prognostication.

The purpose of this study was to evaluate a large number of emergently admitted patients over a 10-year period to pinpoint potential predictors of mortality in patients with a primary diagnosis of blunt chest wall trauma.

## 2. Methods

The aforementioned study compiled data from the National Inpatient Sample repository for patients who were emergently admitted with a primary diagnosis of blunt chest wall trauma (ICD-9 code 959.11) from 2005 to 2014.

The National Inpatient Sample is a government-funded database created by the Agency for Healthcare Research and Quality (AHRQ). AHRQ sponsored the Healthcare Cost and Utilization project to create nationally collected population-based data in standardized formats. Information collected through these large-scale projects allows researchers to evaluate disease outcomes across a multitude of biopsychosocial factors. These analyses can provide valuable insight into clinical outcomes relative to patient demographics, therapeutic interventions, and comorbidities.

The data collected in this study were analyzed and stratified according to parameters of interest, including age differences, sex categories, operation status (operation vs. no operation), and clinical outcomes (survived vs. deceased). Patient attributes were further assessed based upon the following factors: race (white, black, Hispanic, Asian/pacific islander, native American, other), income quartile, insurance status (private insurance, Medicare, Medicaid, self-pay, no charge, other), hospital location (rural, urban non-teaching, urban teaching), other diagnoses (bacterial infections other than TB, respiratory diseases, coagulopathy, cardiac diseases, peripheral vascular diseases, liver disease, genitourinary system diseases, fluid/electrolyte disorders, neoplasms, neurological diseases, platelet/white blood cell diseases, cerebrovascular disease, nonbacterial infections, anemia and/or hemorrhage, digestive diseases other than liver, diabetes, drug abuse, alcohol abuse, tobacco use, hypertension, endocrine disease other than diabetes, nutritional/weight disorders, musculoskeletal and connective tissue disease, psychiatric disease, skin disease, sleep disorders, and trauma, burns, and poisons), invasive diagnostic procedure, surgical procedure, invasive or surgical procedure, hospital length of stay, time to first surgical procedure, and total charges in USD.

Subsequent to stratification, the descriptive and analytical data were utilized to conduct more nuanced analyses demonstrated through tables. The results are presented alongside the average, standard deviation (SD), confidence interval set at 95% (CI), and *p* values (0.05 or less considered significant). The ability to predict mortality based upon individual variables was demonstrated using a univariable logistic regression analysis as well as a multivariable logistic regression analysis. Finally, evaluation of mortality and risk factors were further investigated through a backward logistic regression analysis, in which risk factors were removed via stepwise backward elimination. For these analyses, SPSS version 24 (SPSS Inc., Chicago, IL, USA) and R software (Foundation for Statistical Computing, Vienna, Austria) were used.

The parameters of interest including age differences, race, income quartile, insurance, hospital location, other diagnoses, invasive diagnostic procedure, surgical procedure, invasive or surgical procedure, hospital length of stay, time to first surgical procedure, and total charges in USD are displayed in Table 1. The variables in Table 1 were assessed with a univariable logistic regression for further characterization of differences between male and female patients with blunt chest wall trauma. In addition, the same parameters of interest are displayed in Table 2; however, the variables were further analyzed with a univariable logistic regression for characterization of differences between survived and deceased patients. Similarly, Table 3 utilizes the same parameters of interest and a univariable logistic regression. Table 3 analyses were conducted to determine the differences in patients according to operation status.

The results of a multivariable logistic regression analysis with backward elimination are shown in Table 4. Variables used to standardize outcomes assessed include female sex, social factors, lifestyle behaviors, eating habits, physical exercise, tobacco use, invasive procedure, long term medication usage, alcohol and drug abuse/withdrawal/dependence and other diagnoses. Lastly, Table 5 illustrates data stratified according to survival status for adult and elderly patients with secondary diagnoses further assessed utilizing a univariable regression analysis. The secondary diagnoses included are: observation, tuberculosis, bacterial Infections other than tuberculosis, nonbacterial infections, diabetes, hypertension, anemia and/or hemorrhage, respiratory diseases, coagulopathy, cardiac diseases, cerebrovascular diseases, peripheral vascular diseases, liver diseases, diseases of digestive system other than liver, diseases of oral cavity, salivary glands, and jaws, nutritional/weight disorders, endocrine diseases, genitourinary system diseases, neurological diseases, diseases of the musculoskeletal system and connective tissue, fluid and electrolyte disorders, neoplasms, platelet and white blood cell diseases, psychiatric diseases, skin diseases, trauma, burns and poisoning, drug abuse/withdrawal/dependence, alcohol abuse/withdrawal/dependence, tobacco use, long-term medications/radiotherapy, social factors, sleep disorders, lack of physical exercise, inappropriate diet and eating habits, high risk lifestyle behaviors, body mass index of less than 18.9, body mass index of 19–24.9, body mass index of 25.0–29.9, and body mass index of 30.0 and over. Specific, ICD-9 codes corresponding to secondary diagnoses shown above are displayed in Table 5.

## 3. Results

### 3.1. Age, Gender, Race, and Comorbidity Differences

Between 2005 and 2014, 2158 patients were emergently admitted with a primary diagnosis of blunt chest wall injury. Of these patients, 46.3% were female and 53.6% were male (Table 1). These patients were subdivided into an adult group (age 18–64) and elderly group (age ≥ 65), each of which contained 1127 and 1041 individuals, respectively. The average (SD) age of female adults was 48.5 (12.7) years and male adults was 45.6 (12.3) years. The average (SD) age of elderly females and males studied was 80.3 (8.3) and 77.5 (7.7) years, respectively (Table 1). The majority of adult patients were white, treated in urban teaching hospitals, and possessed private insurance. Elderly patients were overwhelmingly white and were funded by Medicare. Elderly patients were admitted at approximately equal rates in urban teaching and urban non-teaching facilities. Relative rates of comorbidities were categorized according to sex (Table 1). In the adult group, males illustrated higher rates of drug abuse, alcohol abuse, trauma, burns, and poisons. Females exhibited higher rates of bacterial infections (other than TB), anemia, endocrine diseases, and musculoskeletal and connective tissue diagnoses. In the elderly group, males displayed greater rates of coagulopathy, cardiac disease, alcohol abuse, tobacco use, trauma, burns, and poisons, while females presented higher rates of fluid/electrolyte disorders, psychiatric diseases, and musculoskeletal and connective tissue diagnoses (Table 1).

### 3.2. Mortality

In both the adult and elderly groups, 1% of patients died as a result of their blunt chest wall injury (Table 2). Deceased patients in the adult group and elderly group were 10 and 9 years older than their surviving patient counterparts, respectively. Adult patients who passed away exhibited higher rates of the following conditions when compared to surviving adults: respiratory diseases, coagulopathy, peripheral vascular diseases, liver diseases, genitourinary system diseases, fluid/electrolyte disorders, platelet/white blood cell diseases, anemia/hemorrhage, and digestive diseases (Table 2). In contrast, the elderly group displayed no significant differences in comorbidities between the surviving and deceased groups. Adults who died underwent higher rates of invasive diagnostic procedures, surgical procedures, and invasive procedures in comparison to surviving adults. Deceased elderly patients had higher rates of surgical and invasive procedures relative to surviving elderly patients (Table 2). No differences were found between hospital length of stay and time to first surgical procedure between the surviving and deceased groups in either age group prior to stepwise backward elimination.

### 3.3. Operation vs. No Operation

Table 3 represents a stratified analysis regarding operation or lack thereof. In the adult and elderly groups, there were 59 (5%) and 49 (4%) patients that had an operation, respectively. The average age of adults who had operations was 3.6 years older than adults with no operation (Table 3). Elderly patients displayed no significant difference in age between those who had an operation and those who had no operation. Both adult groups were primarily female, white, possessed private insurance, and received treatment at an urban teaching hospital. Both elderly groups were predominantly male, white, funded by Medicare, and received treatment at urban non-teaching hospitals. In comparison to adults who had no operation, adults who underwent an operation displayed higher rates of skin disease, digestive disease, anemia, nonbacterial infections, platelet/WBC diseases, neurological diseases, neoplasms, fluid/electrolyte disorders, genitourinary diseases, liver diseases, peripheral vascular diseases, cardiac disease, coagulopathy, trauma, burns, and poisons (Table 3). Compared to elderly patients who had no operation, elderly patients who underwent surgery demonstrated higher rates of the following diseases: bacterial infections, peripheral vascular diseases, liver diseases, genitourinary diseases, fluid/electrolyte disorders, anemia, diabetes, other endocrine diseases, musculoskeletal and connective tissue disease. In both the adult and elderly groups who underwent an operation, there were higher rates of invasive diagnostic procedures, death, and hospital length of stay, relative to the non-operative counterparts (Table 3).

### 3.4. Risk Factors of Mortality

A backward multivariable logistic regression analysis evaluating associations between mortality and risk factors in patients admitted with primary diagnosis of blunt chest wall injury is displayed in Table 4. Hospital length of stay, liver diseases, respiratory diseases, platelet/white blood cell disease, trauma, burns, and poisons displayed statistically significant associations with mortality. Longer hospital length of stay increased the risk of mortality significantly, OR = 1.09 (95% CI, 1.01–1.18). Age stayed in the final model but showed no statistically significant associations with mortality (Table 4). The presence of trauma, burns, and poisons increased the odds of mortality by more than 3.7 times, while platelet and white blood cell diseases increased the odds of mortality by 4.4 times (Table 4). Lastly, respiratory diseases and liver diseases increased the odds of mortality by 7.4 and 8.3 times, respectively (Table 4).

### 3.5. Lifestyle Habits, Comorbidities and Secondary Diagnoses and Mortality

A stratified analysis based on lifestyle habits, comorbidities and secondary diagnoses is shown in Table 5. Patients were divided into four groups predicated on age and survival. The prevalence of respiratory diseases, peripheral vascular diseases, fluid/electrolyte disorders, diseases of the digestive system, anemia/hemorrhage, coagulopathy, platelet/white blood cell diseases, and genitourinary system diseases was significantly higher in deceased patients than the survived ones (Table 5). Conversely, elderly patients displayed no differences across all lifestyle habits, comorbidities and secondary diagnoses between deceased patients than the survived ones.

## 4. Discussion

### 4.1. Age, Mortality

Elderly and nonelderly adult patients both displayed relatively low mortality rates of 1%. Controlling for a variety of patient characteristics with a multivariable logistic regression analysis and stepwise backward elimination revealed no statistically significant association between mortality and age in patients with blunt chest wall trauma. Our finding is incongruent with previous studies [6,7,8], who found advanced age to be associated with higher rates of mortality. Similarly, Harrington et al. displayed increasing age and injury severity score as independent predictors of survival among patients following blunt chest trauma [9]. Additionally, an 8-year study analyzing prognostic factors in blunt chest trauma revealed age, hemodynamic status, and several comorbidities as the most important survival factors [10]. Contrary to these studies, Vollrath et al. conducted a large-scale study involving 43,289 patients to assess lung failure after polytrauma with concomitant blunt thoracic injury in the elderly. They revealed that lung failure markedly increased hospital length of stay, ICU duration, and mechanical ventilation, independent of age. In addition, lung failure increased mortality significantly more in the oldest patients [11]. These results provide contradicting findings regarding the relationship between age and mortality in blunt chest wall trauma.

Our findings indicate that the relationship between mortality, blunt chest wall trauma, and advanced age may be more nuanced than previously suspected. We presume that the incongruency found in the current literature is due to a lack of control for injury severity, mechanism of blunt chest wall trauma, and severity of comorbidities. Without controlling for patient characteristics, age can serve as a surrogate factor that potentially contains the true mortality-associated variables. We conclude that further investigations controlling for patient characteristics, mechanism of injury, and injury severity are necessary to further elucidate the relationship between age, blunt chest wall trauma, and mortality.

### 4.2. Hospital Length of Stay, Mortality

To the best of our knowledge, relatively limited studies have analyzed the relationship between hospital length of stay (HLOS) and mortality in patients with blunt thoracic trauma. Nevertheless, our findings showed that for every additional day patients with blunt thoracic trauma stayed in the hospital, the odds of mortality increased by 9%. The presence of cardiopulmonary disease and markers of chest wall injury severity such as increased number of rib fractures were associated with increased HLOS and morbidity [12,13,14]. HLOS has been associated with mortality in a variety of acute medical emergencies. More specifically, HLOS has been identified as a major risk factor for mortality in patients emergently hospitalized for hemorrhoids, chronic duodenal ulcers, and ventral hernias [15,16,17,18]. These findings demonstrate the widespread utility of HLOS as a prognosticating tool. Additionally, HLOS is an important predictor of nosocomial infections. Murni et al. conducted a 43-month prospective cohort study that demonstrated that hospital length of stay >7 days significantly increased risk of healthcare-associated infections [19]. This is a concerning finding because multiple large-scale retrospective studies have demonstrated that patients with blunt thoracic traumatic injuries have an HLOS slightly above 9 days on average [20,21].

We suspect that the increased mortality rates observed in patients with increased HLOS may be attributed to nosocomial infections and correspondingly patients with higher acuity who require longer HLOS.

Moving forward, it is critical to identify modifiable clinical and logistical factors to reduce HLOS. An analysis of independent predictors of mortality in patients emergently admitted for arterial embolism and thrombosis concluded that delayed operating room access was significantly associated with mortality and increased HLOS [22]. Furthermore, Levy et al. concluded that increased time to operation was a significantly associated risk factor in mortality for patients emergently admitted for empyema [23]. Operating theater priority may serve as a target to reduce HLOS and mortality in emergent patients. In addition to operational logistics, nutrition has been identified as a critical factor influencing HLOS. Goiburu et al. concluded that malnutrition on admission in trauma patients is an independent risk factor for increased HLOS, morbidity, and mortality [24]. Overall, our results and the supporting literature indicate that increased HLOS should be utilized as an easily accessible prognosticating factor in patients with blunt chest wall trauma. Beyond prognostication, we recommend actively addressing factors influencing HLOS to reduce the risk of nosocomial infections, morbidity, and mortality.

### 4.3. Respiratory Disease, Liver Disease, and Mortality

Beshay et al. assessed 630 patients with blunt chest trauma to determine independent risk factors of mortality [6]. Their study found that the presence of severe lung contusion and advanced age as independent risk factors directly related to mortality involving blunt chest trauma. Similarly, Degirmenci analyzed clinical outcomes in blunt thoracic injuries and concluded that injury severity, associated organ injuries, and pleural and pulmonary parenchymal injuries are the factors most associated with poor clinical outcomes [25]. Furthermore, Battle et al. concluded that an age of 65 years or more, three or more rib fractures, pre-existing cardiopulmonary disease, and post-injury pneumonia were significant risk factors for mortality in patients with blunt chest wall trauma [7].

Our study indicated that patients admitted for blunt chest wall trauma with liver disease and respiratory diseases had significantly higher mortality rates. The presence of respiratory diseases and liver diseases increased the risk of mortality by 7.4 and 8.3 times, respectively. Huber et al. demonstrated that injuries to the lung, advanced age, and heart or thoracic vessel injuries significantly influence mortality after significant blunt chest trauma [26]. Additionally, Perna et al. investigated factors influencing the outcomes in 500 patients with blunt chest trauma and that revealed increased mortality risk was associated with an injury severity score above 25, the presence of three or more rib fractures with flail chest, pulmonary contusion, ARDS, and an age above 55 years [27]. Our study illustrated consistent findings with the current literature regarding an increased risk of mortality in patients with blunt chest wall trauma and respiratory disease.

The increased mortality in patients with blunt chest wall injuries and liver disease seen in our data has not been well assessed in the literature. Furthermore, our results demonstrated that patients with liver disease who experienced blunt chest wall trauma undergo surgery at significantly higher rates. This finding suggests that patients with liver disease possess greater risk of complications as a result of blunt chest wall trauma. We suspect that surgical intervention was warranted at higher rates secondary to attenuated coagulation and immune responses. Coagulative response deficits observed in patients with liver disease have been established through various studies [28,29]. Additionally, coagulative deficits are compounded by large-volume fluid replacement therapy frequently utilized in advanced trauma life support [30,31]. These conclusions require further investigation to determine the mechanism explaining the relationships between clinical outcomes, liver disease, and chest wall trauma. 

### 4.4. Platelet/White Blood Cell Disease, Mortality

Lymphocyte population changes have been established as a potential complication of trauma. However, the exact association between trauma and white blood cell disease has yet to be fully elucidated. Manson et al. conducted an observational cohort study on blunt trauma patients that found evidence of lymphocytic population changes associated with the development of multiple organ dysfunction syndrome and lymphopenia [32]. Koch et al. assessed whether admission with lymphopenia is associated with poor outcomes in patients with blunt chest wall trauma. After controlling for patient demographics, injury severity, and comorbidities, they found no association between lymphopenia and mortality; however, lymphopenia was significantly associated with increased HLOS [33]. In contrast, Vulliamy et al. demonstrated that patients with persistent lymphopenia are 3.5 times more likely to die, and lymphopenia is an independent predictor of increased mortality in patients undergoing emergent general surgery [34]. Similarly, a retrospective study assessing the impact of low lymphocyte count in 2448 blunt trauma patients found that persistently low lymphocyte levels over the first 4 days following moderate and severe injury are associated with increased mortality and increased HLOS [35].

Our results indicated that patients with white blood cell disease were 4.4 times more likely to die as a result of blunt chest wall trauma. These results further contribute to the growing literature describing the association between mortality and white blood cell disease in trauma patients. While the mechanism of action has yet to be elucidated, we suspect that nosocomial infections and increased HLOS are likely key factors influencing these findings. Regardless of the pathogenesis, our findings provide useful information for identifying high-risk patients who may benefit from nonsurgical therapeutic interventions.

### 4.5. Trauma, Burns, Poisons, and Mortality

Severe burns are a clinically challenging ailment to treat that significantly contributes to morbidity and mortality. Fitzwater et al. [36] demonstrated that elevated base deficit at 24 h and septic shock are significantly associated with burn trauma and mortality. Ryan et al. indicated that age, burn size of more than 40% of body surface area, and inhalation injury are the primary risk factors of death after burns [37]. In addition, multiple organ dysfunction syndrome (MODS) has been established as a serious and relatively common complication of severe burn trauma. Nguyen and Nguyen assessed 117 severe-burn adult patients, and of the analyzed patients, 45.3% developed MODS, and 86.79% of those who developed MODS succumbed to their injuries [38]. Our results are consistent with the current literature, specifically illustrating that patients admitted for blunt chest wall trauma with burns were 3.72-fold more likely to pass away as a result of their trauma. These findings emphasize the importance of rapid therapeutic intervention in the case of blunt chest wall trauma with concomitant burns. 

### 4.6. Operation, Non-Operation, and Mortality

The decision to provide operative care to patients with blunt thoracic trauma has increased in prevalence in recent years [39]. Ingoe et al. conducted a retrospective analysis of 18,000 patients with blunt thoracic trauma to determine the strongest predictors of surgical rib fracture fixation. They found that older age, unilateral flail chest injury, major trauma center treatment, and male gender were all significant predictors of surgical rib fixation [40]. Granetzny et al. compared clinical outcomes in surgically versus conservatively treated patients with flail chest. Their results demonstrated significantly improved chest wall stability and pulmonary function in operated patients relative to their non-operative counterparts [41]. These studies illustrate potential predictors of surgical interventions as well as the therapeutic value of these operations in patients with blunt chest trauma.

Our comparison of operatively and non-operatively managed adult patients with blunt chest trauma demonstrated significantly higher mortality rates, longer hospital lengths of stay, and older age in the operatively managed adult group. Moreover, operatively managed elderly patients with blunt chest trauma displayed higher mortality rates and longer hospital length of stay in comparison to non-operative elderly patients. The necessity for operation could be an index of severity of blunt chest trauma, which caused higher mortality and longer HLOS in both adults and elderly patients. In addition, adult and elderly patients who received operative care displayed higher rates of peripheral vascular disease, liver disease, genitourinary system diseases, fluid and electrolyte disorders, and anemia/hemorrhage relative to their respective non-operative counterparts. These findings contribute to previous studies elucidating the selecting features for operative care in adult and elderly patients with blunt chest trauma. We recommend further investigation into clinical factors utilized to determine when operative versus non-operative care is appropriate in patients with blunt chest wall trauma. To adequately determine when operative intervention is appropriate, an analysis of clinical outcomes relative to surgical techniques, comorbidities, chest wall trauma severity, and presence of surgically amendable injuries is required. These considerations were outside the scope of this study.

### 4.7. Coagulopathy, Mortality

Coagulopathy has been established as a major consequence of trauma [42,43,44]. In our study, coagulopathy significantly increased mortality in patients emergently admitted with blunt chest wall trauma. This finding is supported by Macleod et al., who conducted a retrospective trauma study including greater than 10,000 patients that showed that prothrombin time and partial thromboplastin time independently predicted all-cause mortality [45]. Floccard et al. elucidated the pathogenic mechanisms involved in trauma-induced coagulopathy as demonstrated by reductions in protein C activity and factor V early after injury [46]. Maegele et al. revealed that acute traumatic coagulopathy is associated with increased morbidity and mortality and is seen in one of four patients with major trauma [47]. Brohi et al. demonstrated coagulopathy results in increased transfusion requirements, organ dysfunction, critical care stay, and mortality [48]. Our findings and supporting literature suggest that patients admitted with blunt chest trauma require immediate coagulopathic interventions to address the increased mortality associated with trauma-induced coagulopathy.

### 4.8. Strengths of the Study

The data included in our study are part of a nationally representative sample because of the large sample size collected from multiple hospitals across the United States. Furthermore, a wide variety of patient and hospital characteristics were incorporated. The diversity of information allowed for the standardization of clinical information and sophisticated analyses of biological and sociological markers. Therefore, statistically sound conclusions were obtained from our study that can be generalized to a wide variety of patients. Lastly, our study introduces potential opportunities for further investigations, particularly regarding the relationships between age, respiratory disease, HLOS, burns, liver disease, mortality, and blunt chest trauma.

### 4.9. Limitations of the Study

Our study does not include information regarding types of blunt chest wall trauma, severity of injuries, etiology of trauma, time to operation, operative techniques, and comorbidity severity parameters. Future research involving specific chest wall injuries, interventional approaches, and severity of comorbidities could shed light on the nuances discussed above.

## 5. Conclusions

Patients emergently admitted for blunt chest trauma with longer hospital length of stay, pulmonary disease, liver disease, white blood cell disease, and/or burns exhibited higher odds of mortality rates. Every additional day blunt chest wall trauma patients stay in the hospital correlates to a 9% increased risk of mortality. Age is not associated with mortality in patients emergently admitted for blunt chest trauma.

## Figures and Tables

**Table 1:** Characteristics of emergently admitted patients with a primary diagnosis of blunt chest wall injury. Data are stratified according to sex categories, NIS 2004–2014.

		Adult, N (%)			Elderly, N (%)		
		Male	Female	*P*	Male	Female	*P*
**All Cases**		721 (64)	399 (36)		438 (42)	601 (58)	
Race	White	329 (55)	181 (53)	0.910	287 (76)	385 (76)	0.480
	Black	117 (20)	74 (22)		23 (6)	37 (7)	
	Hispanic	103 (17)	54 (16)		46 (12)	50 (10)	
	Asian/Pacific Islander	17 (3)	13 (4)		8 (2)	15 (3)	
	Native American	4 (1)	2 (1)		0 (0)	3 (1)	
	Other	27 (4)	15 (4)		12 (3)	14 (3)	
Income Quartile	Quartile 1	206 (31)	123 (33)	0.310	117 (28)	145 (25)	0.260
	Quartile 2	168 (25)	77 (21)		118 (28)	151 (26)	
	Quartile 3	146 (22)	94 (25)		91 (22)	133 (23)	
	Quartile 4	145 (22)	80 (21)		94 (22)	161 (27)	
Insurance	Private Insurance	291 (40)	196 (49)	**<0.001**	115 (26)	124 (21)	0.280
	Medicare	87 (12)	52 (13)		296 (68)	444 (74)	
	Medicaid	105 (15)	70 (18)		8 (2)	11 (2)	
	Self-Pay	138 (19)	54 (14)		9 (2)	9 (2)	
	No Charge	12 (2)	5 (1)		0 (0)	1 (0.2)	
	Other	87 (12)	20 (5)		9 (2)	10 (2)	
Hospital Location	Rural	90 (13)	50 (13)	0.320	66 (15)	91 (15)	0.490
	Urban: Non-Teaching	271 (38)	133 (33)		209 (48)	267 (45)	
	Urban: Teaching	356 (50)	215 (54)		160 (37)	241 (40)	
Other Diagnoses	Bacterial Infections (Other than TB)	1 (0.1)	10 (3)	**<0.001**	11 (3)	26 (4)	0.120
	Respiratory Diseases	260 (36)	146 (37)	0.860	219 (50)	303 (50)	0.900
	Coagulopathy	22 (3)	14 (4)	0.680	60 (14)	48 (8)	**0.003**
	Cardiac Diseases	237 (33)	114 (29)	0.140	288 (66)	333 (55)	**<0.001**
	Peripheral Vascular Diseases	30 (4)	14 (4)	0.590	37 (8)	48 (8)	0.790
	Liver Diseases	33 (5)	18 (5)	0.960	8 (2)	12 (2)	0.840
	Genitourinary System Diseases	80 (11)	57 (14)	0.120	141 (32)	160 (27)	0.051
	Fluid and Electrolyte Disorders	74 (10)	35 (9)	0.420	74 (17)	137 (23)	**0.020**
	Neoplasms	26 (4)	22 (6)	0.130	70 (16)	84 (14)	0.370
	Neurological Diseases	79 (11)	64 (16)	**0.015**	113 (26)	154 (26)	0.950
	Platelet and White Blood Cell Diseases	18 (3)	11 (3)	0.790	21 (5)	27 (5)	0.820
	Trauma, Burns, and Poisons	314 (44)	165 (41)	0.480	172 (39)	191 (32)	**0.012**
	Cerebrovascular Diseases	10 (1)	7 (2)	0.630	25 (6)	23 (4)	0.150
	Nonbacterial Infections	32 (4)	15 (4)	0.590	6 (1)	12 (2)	0.440
	Anemia and/or Hemorrhage	46 (6)	39 (10)	**0.040**	65 (15)	87 (15)	0.870
	Digestive Diseases other than Liver	83 (12)	65 (16)	**0.024**	111 (25)	176 (29)	0.160
	Diabetes	111 (15)	81 (20)	**0.037**	125 (29)	140 (23)	0.060
	Drug Abuse	71 (10)	25 (6)	**0.040**	3 (1)	7 (1)	0.430
	Alcohol Abuse	135 (19)	26 (7)	**<0.001**	25 (6)	6 (1)	**<0.001**
	Tobacco Use	171 (24)	80 (20)	0.160	75 (17)	55 (9)	**<0.001**
	Hypertension	270 (37)	153 (38)	0.770	287 (66)	412 (69)	0.300
	Endocrine Diseases other than Diabetes	122 (17)	111 (28)	**<0.001**	152 (35)	241 (40)	0.080
	Nutritional/Weight Disorders	181 (25)	98 (25)	0.840	175 (40)	241 (40)	0.960
	Musculoskeletal and Connective Tissue	152 (21)	120 (30)	**<0.001**	140 (32)	286 (48)	**<0.001**
	Psychiatric Diseases	106 (15)	63 (16)	0.630	54 (12)	110 (18)	**0.009**
	Skin Diseases	18 (3)	15 (4)	0.230	19 (4)	25 (4)	0.890
	Sleep Disorders	16 (2)	16 (4)	0.090	23 (5)	21 (4)	0.170
Invasive Diagnostic Procedure		28 (4)	8 (2)	0.090	8 (2)	8 (1)	0.520
Surgical Procedure		40 (6)	19 (5)	0.570	14 (3)	25 (4)	0.420
Invasive or Surgical Procedure		63 (9)	25 (6)	0.140	21 (5)	31 (5)	0.790
Deceased		5 (0.7)	3 (0.8)	0.910	4 (0.9)	3 (0.5)	0.420
		**Mean (SD)**	**Mean (SD)**	***P***	**Mean (SD)**	**Mean (SD)**	***P***
Age, Years		45.6 (12.3)	48.5 (12.7)	**<0.001**	77.5 (7.7)	80.0 (8.3)	**<0.001**
Time to First Surgical Procedure, Days		1.5 (2.2)	1.4 (1.0)	0.760	2.0 (3.4)	1.8 (2.1)	0.800
Hospital Length of Stay, Days		2.0 (2.4)	2.2 (2.5)	0.210	2.9 (3.2)	2.9 (2.7)	0.870
Total Charges, USD		21,040 (47,799)	19,726 (19,851)	0.600	20,882 (25,266)	17,711 (17,252)	0.025

Bold indicates result is statistically significant.

**Table 2:** Characteristics of emergently admitted patients with a primary diagnosis of blunt chest wall injury. Data are classified according to outcome categories, NIS 2004–2014.

		Adult, N (%)			Elderly, N (%)		
		Survived	Deceased	*P*	Survived	Deceased	*P*
**All Cases**		1119 (99)	8 (1)		1034 (99)	7 (1)	
Sex, Female		396 (36)	3 (36)	0.999	598 (58)	3 (43)	0.460
Race	White	507 (54)	3 (75)	0.999	668 (76)	4 (80)	0.560
	Black	190 (20)	1 (25)		59 (7)	1 (20)	
	Hispanic	157 (17)	0 (0)		96 (11)	0 (0)	
	Asian/Pacific Islander	30 (3)	0 (0)		23 (3)	0 (0)	
	Native American	6 (1)	0 (0)		3 (0.3)	0 (0)	
	Other	42 (5)	0 (0)		26 (3)	0 (0)	
Income Quartile	Quartile 1	329 (32)	3 (43)	0.620	260 (26)	2 (29)	0.999
	Quartile 2	244 (24)	2 (29)		267 (27)	2 (29)	
	Quartile 3	239 (23)	2 (29)		224 (22)	1 (14)	
	Quartile 4	227 (22)	0 (0)		254 (25)	2 (29)	
Insurance	Private Insurance	488 (44)	4 (50)	0.590	239 (23)	1 (14)	0.060
	Medicare	136 (12)	2 (25)		737 (72)	4 (57)	
	Medicaid	175 (16)	0 (0)		18 (2)	1 (14)	
	Self-Pay	191 (17)	2 (25)		17 (2)	1 (14)	
	No Charge	17 (2)	0 (0)		1 (0.1)	0 (0)	
	Other	109 (10)	0 (0)		19 (2)	0 (0)	
Hospital Location	Rural	140 (13)	0 (0)	0.340	157 (15)	0 (0)	0.640
	Urban: Non-Teaching	404 (36)	5 (63)		473 (46)	4 (67)	
	Urban: Teaching	570 (51)	3 (37)		400 (39)	2 (33)	
Other Diagnoses	Bacterial Infections (Other than TB)	10 (1)	1 (13)	0.080	36 (4)	1 (14)	0.220
	Respiratory Diseases	398 (36)	7 (88)	**0.004**	516 (50)	6 (86)	0.120
	Coagulopathy	33 (3)	3 (38)	**0.002**	108 (10)	0 (0)	0.999
	Cardiac Diseases	347 (31)	5 (63)	0.120	616 (60)	5 (71)	0.710
	Peripheral Vascular Diseases	42 (4)	2 (25)	**0.036**	85 (8)	0 (0)	0.999
	Liver Diseases	48 (4)	3 (38)	**0.004**	19 (2)	1 (14)	0.130
	Genitourinary System Diseases	133 (12)	4 (50)	**0.010**	298 (29)	3 (43)	0.420
	Fluid and Electrolyte Disorders	105 (9)	4 (50)	**0.004**	209 (20)	2 (29)	0.640
	Neoplasms	47 (4)	1 (13)	0.300	153 (15)	1 (14)	0.999
	Neurological Diseases	140 (13)	3 (38)	0.070	266 (26)	1 (14)	0.690
	Platelet and White Blood Cell Diseases	26 (2)	3 (38)	**<0.001**	48 (5)	0 (0)	0.999
	Trauma, Burns, and Poisons	479 (43)	5 (63)	0.300	358 (35)	5 (71)	0.054
	Cerebrovascular Diseases	17 (2)	0 (0)	0.999	47 (5)	1 (14)	0.280
	Nonbacterial Infections	47 (4)	0 (0)	0.999	18 (2)	0 (0)	0.999
	Anemia and/or Hemorrhage	82 (7)	3 (38)	**0.018**	152 (15)	0 (0)	0.600
	Digestive Diseases other than Liver	143 (13)	5 (63)	**0.001**	286 (28)	1 (14)	0.680
	Diabetes	190 (17)	2 (25)	0.630	262 (25)	3 (43)	0.380
	Drug Abuse	95 (9)	0 (0)	0.999	9 (1)	1 (14)	0.070
	Alcohol Abuse	158 (14)	3 (38)	0.090	30 (3)	1 (14)	0.190
	Tobacco Use	248 (22)	2 (25)	0.999	130 (13)	0 (0)	0.610
	Hypertension	421 (38)	1 (13)	0.270	696 (67)	3 (43)	0.230
	Endocrine Diseases other than Diabetes	230 (21)	3 (38)	0.220	389 (38)	4 (57)	0.440
	Nutritional/Weight Disorders	276 (25)	3 (38)	0.420	415 (40)	1 (14)	0.250
	Musculoskeletal and Connective Tissue	269 (24)	3 (38)	0.410	422 (41)	4 (57)	0.450
	Psychiatric Diseases	168 (15)	1 (13)	0.999	164 (16)	0 (0)	0.610
	Skin Diseases	32 (3)	1 (13)	0.210	44 (4)	0 (0)	0.999
	Sleep Disorders	32 (3)	0 (0)	0.999	44 (4)	0 (0)	0.999
Invasive Diagnostic Procedure		34 (3)	2 (25)	**0.025**	16 (2)	0 (0)	0.999
Surgical Procedure		53 (5)	6 (75)	**<0.001**	37 (4)	2 (29)	**0.026**
Invasive or Surgical Procedure		81 (7)	7 (88)	**<0.001**	50 (5)	2 (29)	**0.044**
		**Mean (SD)**	**Mean (SD)**	***P***	**Mean (SD)**	**Mean (SD)**	***P***
Age, Years		46.4 (12.6)	56.4 (5.4)	**0.001**	78.9 (8.1)	87.9 (10.4)	**0.004**
Time to First Surgical Procedure, Days		1.4 (1.6)	3.0 (4.4)	0.580	1.8 (2.6)	3.0 (4.2)	0.530
Hospital Length of Stay, Days		2.0 (2.3)	8.4 (7.7)	0.051	2.9 (2.9)	3.7 (3.1)	0.460
Total Charges, USD		19,898 (35,917)	122,169 (199,237)	0.190	19,013 (21,062)	23,053 (19,135)	0.610

Bold indicates result is statistically significant.

**Table 3:** Characteristics of emergently admitted patients with a primary diagnosis of blunt chest wall injury. Data are stratified according to operation status, NIS 2004–2014.

		Adult, N (%)			Elderly, N (%)		
		No Operation	Operation	*P*	No Operation	Operation	*P*
**All Cases**		1069 (95)	59 (5)		1002 (96)	39 (4)	
Sex, Female		380 (36)	19 (32)	0.570	576 (58)	25 (64)	0.420
Race	White	481 (54)	29 (63)	0.650	652 (77)	20 (61)	**0.029**
	Black	184 (21)	8 (17)		57 (7)	3 (9)	
	Hispanic	149 (17)	8 (17)		86 (10)	10 (30)	
	Asian/Pacific Islander	30 (3)	0 (0)		23 (3)	0 (0)	
	Native American	6 (1)	0 (0)		3 (0.4)	0 (0)	
	Other	41 (5)	1 (2)		26 (3)	0 (0)	
Income Quartile	Quartile 1	316 (32)	17 (32)	0.660	253 (26)	9 (23)	0.750
	Quartile 2	230 (23)	16 (30)		256 (26)	13 (33)	
	Quartile 3	229 (23)	12 (22)		216 (22)	9 (23)	
	Quartile 4	218 (22)	9 (17)		248 (26)	8 (21)	
Insurance	Private Insurance	472 (44)	20 (35)	**<0.001**	227 (23)	13 (33)	0.510
	Medicare	122 (11)	17 (29)		716 (72)	25 (64)	
	Medicaid	162 (15)	13 (22)		19 (2)	0 (0)	
	Self-Pay	187 (18)	6 (10)		18 (2)	0 (0)	
	No Charge	16 (2)	1 (2)		1 (0.1)	0 (0)	
	Other	108 (10)	1 (2)		18 (2)	1 (3)	
Hospital Location	Rural	133 (13)	7 (12)	0.570	155 (16)	2 (5)	0.180
	Urban: Non-Teaching	391 (37)	18 (31)		456 (46)	21 (54)	
	Urban: Teaching	540 (51)	34 (58)		386 (39)	16 (41)	
Other Diagnoses	Bacterial Infections (Other than TB)	9 (1)	2 (3)	0.110	32 (3)	5 (13)	**0.010**
	Respiratory Diseases	379 (36)	27 (46)	0.110	500 (50)	22 (56)	0.430
	Coagulopathy	31 (3)	5 (9)	**0.018**	104 (10)	4 (10)	0.999
	Cardiac Diseases	322 (30)	30 (51)	**<0.001**	592 (59)	29 (74)	0.060
	Peripheral Vascular Diseases	32 (3)	12 (20)	**<0.001**	75 (8)	10 (26)	**<0.001**
	Liver Diseases	45 (4)	6 (10)	**0.032**	17 (2)	3 (8)	**0.036**
	Genitourinary System Diseases	113 (11)	24 (41)	**<0.001**	275 (27)	26 (67)	**<0.001**
	Fluid and Electrolyte Disorders	94 (9)	15 (25)	**<0.001**	196 (20)	15 (39)	**0.004**
	Neoplasms	42 (4)	6 (10)	**0.021**	147 (15)	7 (18)	0.570
	Neurological Diseases	125 (12)	18 (31)	**<0.001**	255 (25)	12 (31)	0.460
	Platelet and White Blood Cell Diseases	21 (2)	8 (14)	**<0.001**	47 (5)	1 (3)	0.999
	Trauma, Burns, and Poisons	451 (42)	33 (56)	**0.038**	347 (35)	16 (41)	0.410
	Cerebrovascular Diseases	16 (2)	1 (2)	0.600	45 (5)	3 (8)	0.420
	Nonbacterial Infections	41 (4)	6 (10)	**0.018**	16 (2)	2 (5)	0.140
	Anemia and/or Hemorrhage	68 (6)	17 (29)	**<0.001**	136 (14)	16 (41)	**<0.001**
	Digestive Diseases other than Liver	131 (12)	17 (29)	**<0.001**	278 (28)	9 (23)	0.520
	Diabetes	179 (17)	13 (22)	0.290	248 (25)	17 (44)	**0.008**
	Drug Abuse	88 (8)	8 (14)	0.150	9 (1)	1 (3)	0.320
	Alcohol Abuse	148 (14)	13 (22)	0.080	31 (3)	0 (0)	0.630
	Tobacco Use	233 (22)	18 (31)	0.120	125 (13)	5 (13)	0.950
	Hypertension	397 (37)	26 (44)	0.280	673 (67)	26 (67)	0.950
	Endocrine Diseases other than Diabetes	217 (20)	16 (27)	0.210	371 (37)	22 (56)	**0.014**
	Nutritional/Weight Disorders	261 (24)	18 (31)	0.290	402 (40)	14 (36)	0.600
	Musculoskeletal and Connective Tissue	259 (24)	13 (22)	0.700	416 (42)	10 (26)	**0.048**
	Psychiatric Diseases	161 (15)	8 (14)	0.750	159 (16)	5 (13)	0.610
	Skin Diseases	27 (3)	6 (10)	**<0.001**	42 (4)	2 (5)	0.680
	Sleep Disorders	31 (3)	1 (2)	0.999	43 (4)	1 (3)	0.999
Invasive Diagnostic Procedure		29 (3)	7 (12)	**<0.001**	13 (1)	3 (8)	**0.019**
Deceased		2 (0.2)	6 (10)	**<0.001**	5 (0.5)	2 (5)	**0.026**
		**Mean (SD)**	**Mean (SD)**	***p***	**Mean (SD)**	**Mean (SD)**	***p***
Age, Years		46.3 (12.6)	49.9 (10.5)	**0.016**	78.9 (8.2)	79.7 (7.8)	0.540
Hospital Length of Stay, Days		1.9 (1.7)	6.0 (6.8)	**<0.001**	2.8 (2.7)	5.5 (5.5)	**0.004**
Total Charges, USD		17,834 (15,174)	70,634 (154,924)	**0.011**	18,178 (18,801)	40,826 (47,568)	**0.005**

Bold indicates result is statistically significant.

**Table 4:** Backward multivariable logistic regression analysis to evaluate the associations between mortality and different risk factors in patients emergently admitted with a primary diagnosis of blunt chest wall injury (NIS 2004–2014). Mortality is the dependent variable.

Patient Characteristics	Mortality	
	N = 2158	R^2^ = 0.229
	OR (95% CI)	*P*
Number of Events	N = 15	
Hospital Length of Stay, Days	1.09 (1.01, 1.18)	**0.033**
Age, Years	1.03 (0.99, 1.07)	0.100
Liver Diseases	8.36 (2.23, 31.37)	**0.002**
Respiratory Diseases	7.46 (1.63, 34.11)	**0.010**
Trauma, Burns, and Poisons	3.72 (1.18, 11.71)	**0.025**
Platelet and White Blood Cell Diseases	4.42 (1.09, 17.91)	**0.038**
Invasive Procedure	Removed Via Stepwise Backward Elimination	
Sex, Female		
Bacterial Infections (Other than Tuberculosis)		
Coagulopathy		
Cardiac Diseases		
Peripheral Vascular Diseases		
Genitourinary System Diseases		
Fluid and Electrolyte Disorders		
Neoplasms		
Neurological Diseases		
Cerebrovascular Diseases		
Tuberculosis		
Nonbacterial Infections		
Anemia and/or Hemorrhage		
Digestive Diseases other than Liver		
Diabetes		
Drug Abuse/Withdrawal/Dependence		
Alcohol Abuse/Withdrawal/Dependence		
Tobacco Use		
Hypertension		
Endocrine Diseases other than Diabetes		
Nutritional/Weight Disorders		
Musculoskeletal System and Connective Tissue Diseases		
Psychiatric Diseases		
Skin Diseases		
Long Term Medication Usage		
Diseases of Oral Cavity, Salivary Glands, and Jaw		
Sleep Disorders		
Lack of Physical Exercise		
Inappropriate Diet and Eating Habits		
High Risk Lifestyle Behaviors		
Social Factors		

Bold indicates result is statistically significant.

**Table 5:** Secondary diagnoses of patients emergently admitted with a primary diagnosis of blunt chest wall injury (NIS 2004–2014). Data are stratified according to survival status.

	Adult, N (%)			Elderly, N (%)		
Lifestyle Habits, Comorbidities and Secondary Diagnoses (ICD-9 Codes)	Survived	Deceased	*p* Value	Survived	Deceased	*p* Value
Observations	1119 (99)	8 (1)		1034 (99)	7 (1)	
Tuberculosis (010.0–018.96)	0 (0)	0 (0)		1 (0.1)	0 (0)	0.999
Bacterial Infections Other than Tuberculosis (020.0–041.9, 790.7)	10 (1)	1 (13)	0.080	36 (4)	1 (14)	0.220
Nonbacterial Infections (042, 795.71, V08, 045.0–139.8, 790.8, and/or presence of Comorbidity of AIDS)	47 (4)	0 (0)	0.999	18 (2)	0 (0)	0.999
Diabetes (250.0–250.93, V58.67, and/or presence of Comorbidity of Diabetes Uncomplicated or Diabetes Chronic Complications)	190 (17)	2 (25)	0.630	262 (25)	3 (43)	0.380
Hypertension (401.0–405.99, 796.2, and/or presence of Comorbidity of Hypertension)	421 (38)	1 (13)	0.270	696 (67)	3 (43)	0.230
Anemia and/or Hemorrhage (280.0–285.9, 784.7, 784.8, and/or presence of Comorbidity of Anemia)	82 (7)	3 (38)	**0.018**	152 (15)	0 (0)	0.600
Respiratory Diseases (415.0–417.9, 460–519.9, 784.91, 786, and/or presence of Comorbidity of COPD, ILD or Pulmonary Circulation Disease)	398 (36)	7 (88)	**0.004**	516 (50)	1 (14)	0.120
Coagulopathy (286.0–286.9, 790.92, V58.61, V58.63, and/or presence of Comorbidity of Coagulopathy)	33 (3)	3 (38)	**0.002**	108 (10)	0 (0)	0.999
Cardiac Diseases (391.X, 392.0, 393.398.99, 410.0–414.9, 420.0–429.9, 794.3X, 785.XX, and/or presence of Comorbidity of CHF or Valvular Diseases)	347 (31)	5 (63)	0.120	616 (60)	5 (71)	0.710
Cerebrovascular Diseases (325, 430–438)	17 (2)	0 (0)	0.999	47 (5)	1 (14)	0.280
Peripheral Vascular Diseases (440–457.9, and/or presence of Comorbidity of Peripheral Vascular Disorders)	42 (4)	2 (25)	**0.036**	85 (8)	0 (0)	0.999
Liver Diseases (570–573.9, 790.4, 794.8, and/or presence of Comorbidity of Liver Diseases)	48 (4)	3 (38)	**0.004**	19 (2)	1 (14)	0.130
Diseases of Digestive System other than Liver (530.00–569.9, 574.0–579.9, 787, 001.0–009.3, and/or presence of Comorbidity of Peptic Ulcer)	143 (13)	5 (63)	**0.001**	286 (28)	1 (14)	0.680
Diseases of Oral Cavity, Salivary Glands, and Jaws (520–529)	2 (0.2)	0 (0)	0.999	4 (0.4)	0 (0)	0.999
Nutritional/Weight Disorders (260–273.9, 275.XX,277.0–278.8, 783.XX, 799.3–799.4, and/or presence of Comorbidity of Weight Loss)	276 (25)	3 (38)	0.420	415 (40)	1 (14)	0.250
Endocrine Diseases other than Diabetes (240.0–259.9, 991.0–992.9, and/or presence of Comorbidity of Endocrine Diseases)	230 (21)	3 (38)	0.220	389 (38)	4 (57)	0.440
Genitourinary System Diseases (580.0–629.9, 403.XX, 791.XX, 788.XX, and/or presence of Comorbidity of Renal Diseases)	133 (12)	4 (50)	**0.010**	298 (29)	3 (43)	0.420
Neurological Diseases (317.0–326, 330.0–337.9, 340–359.9, 392, 780.0–780.09, 780.2–780.4, 317–319, 290.XX, 294.XX, 781.0–782.0, and/or presence of Comorbidity of Paralysis or Other Neurological Disorders or Paralysis)	140 (13)	3 (38)	0.070	266 (26)	1 (14)	0.690
Diseases of the Musculoskeletal System and Connective Tissue (274.XX, 710.0–739, and/or presence of Comorbidity of Rheumatoid Arthritis or Lupus)	269 (24)	3 (38)	0.410	422 (41)	4 (57)	0.450
Fluid and Electrolyte Disorders (275.0–276.9, 458.0–459.9, and/or presence of Comorbidity of Fluid and Electrolyte Disorders)	105 (9)	4 (50)	**0.004**	209 (20)	2 (29)	0.640
Neoplasms (140.0–239.9, V10.XX, and/or presence of Comorbidity of Lymphoma, Metastatic Diseases, or Tumor)	47 (4)	1 (13)	0.300	153 (15)	1 (14)	0.999
Platelet and White Blood Cell Diseases (204.0–208.92, 287.0–288.9, 238.71)	26 (2)	3 (38)	**<0.001**	48 (5)	0 (0)	0.999
Psychiatric Diseases (293.XX, 295.0–302.9, 306.0–316, 780.1, V62.8, V15.4, and/or presence of Comorbidity of Psychoses)	168 (15)	1 (13)	0.999	164 (16)	0 (0)	0.610
Skin Diseases (680.0–709.9, 782.1–782.9)	32 (3)	1 (13)	0.210	44 (4)	0 (0)	0.999
Trauma, Burns and Poisoning (800–999)	479 (43)	5 (63)	0.300	358 (35)	5 (71)	0.054
Drug Abuse/Withdrawal/Dependence (292.0–292.9, 304.0–304.93, 305.2–305.93, and/or presence of Comorbidity of Drug Abuse)	95 (9)	0 (0)	0.999	9 (1)	1 (14)	0.070
Alcohol Abuse/Withdrawal/Dependence (291.0–291.9, 303.0–303.93, 305.0–305.03, and/or presence of Comorbidity of Alcohol Abuse)	158 (14)	3 (38)	0.090	30 (3)	1 (14)	0.190
Tobacco Use (305.1)	248 (22)	22 (25)	0.999	130 (13)	0 (0)	0.610
Long-Term Medications/Radiotherapy (V58.0-V58.2, V58.62, V58.64-V58.66, V58.68-V58.69)	41 (4)	0 (0)	0.999	92 (9)	0 (0)	0.999
Social Factors (V60.0-V62.6, V63.0-V64.3, V15.81)	43 (4)	0 (0)	0.999	22 (2)	0 (0)	0.999
Sleep Disorders (327, 780.5, V69.4, V69.5)	32 (3)	0 (0)	0.999	44 (4)	0 (0)	0.999
Lack of Physical Exercise (V69.0)	0 (0)	0 (0)		1 (0.1)	0 (0)	0.999
Inappropriate Diet and Eating Habits (V69.1)	0 (0)	0 (0)		0 (0)	0 (0)	
High Risk Lifestyle Behaviors (V69.2, V69.3)	0 (0)	0 (0)		0 (0)	0 (0)	
Body Mass Index of Less than 18.9 (V85.0)	2 (10)	0 (0)		2 (12)	0 (0)	
Body Mass Index of 19–24.9 (V85.1)	0 (0)	0 (0)		5 (29)	0 (0)	
Body Mass Index of 25.0–29.9 (V85.21-V85.25)	0 (0)	0 (0)		0 (0)	0 (0)	
Body Mass Index of 30.0 and over (V85.30-V85.45)	19 (90)	0 (0)		10 (59)	0 (0)	

Bold indicates result is statistically significant.

## Data Availability

National inpatient sample database can be found on the Healthcare Cost & Utilization Project website through the following URL, https://www.hcup-us.ahrq.gov/db/nation/nis/nisdbdocumentation.jsp, accessed on 14 March 2022.
